# pYR4 From a Norwegian Isolate of *Yersinia ruckeri* Is a Putative Virulence Plasmid Encoding Both a Type IV Pilus and a Type IV Secretion System

**DOI:** 10.3389/fcimb.2018.00373

**Published:** 2018-10-30

**Authors:** Agnieszka Wrobel, Claudio Ottoni, Jack C. Leo, Dirk Linke

**Affiliations:** ^1^Department of Biosciences, University of Oslo, Oslo, Norway; ^2^Centre for Ecological and Evolutionary Synthesis, University of Oslo, Oslo, Norway

**Keywords:** *tra* operon, *pil* operon, conjugative plasmid, *Yersinia ruckeri*, type IV secretion system

## Abstract

Enteric redmouth disease caused by the pathogen *Yersinia ruckeri* is a significant problem for fish farming around the world. Despite its importance, only a few virulence factors of *Y. ruckeri* have been identified and studied in detail. Here, we report and analyze the complete DNA sequence of pYR4, a plasmid from a highly pathogenic Norwegian *Y. ruckeri* isolate, sequenced using PacBio SMRT technology. Like the well-known pYV plasmid of human pathogenic *Yersiniae*, pYR4 is a member of the IncFII family. Thirty-one percent of the pYR4 sequence is unique compared to other *Y. ruckeri* plasmids. The unique regions contain, among others genes, a large number of mobile genetic elements and two partitioning systems. The G+C content of pYR4 is higher than that of the *Y. ruckeri* NVH_3758 genome, indicating its relatively recent horizontal acquisition. pYR4, as well as the related plasmid pYR3, comprises operons that encode for type IV pili and for a conjugation system (*tra*). In contrast to other *Yersinia* plasmids, pYR4 cannot be cured at elevated temperatures. Our study highlights the power of PacBio sequencing technology for identifying mis-assembled segments of genomic sequences. Comparative analysis of pYR4 and other *Y. ruckeri* plasmids and genomes, which were sequenced by second and the third generation sequencing technologies, showed errors in second generation sequencing assemblies. Specifically, in the *Y. ruckeri* 150 and *Y. ruckeri* ATCC29473 genome assemblies, we mapped the entire pYR3 plasmid sequence. Placing plasmid sequences on the chromosome can result in erroneous biological conclusions. Thus, PacBio sequencing or similar long-read methods should always be preferred for *de novo* genome sequencing. As the *tra* operons of pYR3, although misplaced on the chromosome during the genome assembly process, were demonstrated to have an effect on virulence, and type IV pili are virulence factors in many bacteria, we suggest that pYR4 directly contributes to *Y. ruckeri* virulence.

## Introduction

The genus *Yersinia* consists of 17 different species (Reuter et al., 2014; Savin et al., 2014). Although the human pathogens within the genus are closely related to each other, they cause diverse diseases. *Y. pestis*, the causative agent of bubonic and pneumonic plague, is one of the most virulent organisms known (Chauhan et al., 2016). In addition, this genus includes *Y. enterocolitica* and *Y. pseudotuberculosis*, well-known human enteropathogens. *Y. pestis* spreads through fleabites or aerosols, whereas *Y. enterocolitica* and *Y. pseudotuberculosis* are transmitted via ingestion of contaminated food or water (Bottone, 1997; Perry and Fetherston, 1997; Jalava et al., 2006). *Y. enterocolitica* and *Y. pseudotuberculosis* are responsible for a broad range of diseases ranging from mild gastroenteritis to life-threatening septicemia (Bottone, 1997).

*Y. ruckeri* is a fish pathogen causing enteric redmouth disease (ERM), mainly in salmonids (Bullock et al., 1978; Busch, 1978). This bacterium contributes to enormous economic losses in aquaculture throughout the world. *Y. ruckeri* is mostly transmitted through contact with carrier fish (Busch, 1978; Stevenson and Airdrie, 1984). Despite the availability of vaccines, yersiniosis outbreaks still occur in fish farms (Ormsby et al., 2016). The majority of the ERM outbreaks are caused by the highly pathogenic *Y. ruckeri* serotype 1 belonging to biotype 1, characterized as motile with phospholipase activity (Romalde and Toranzo, 1993). For a long time, ERM has played a minor role in Norway, with only a few outbreaks per year (Hjeltnes et al., 2017). The first report of a disease outbreak caused by *Y. ruckeri* among Atlantic salmon was described in Norway in 1985, and this was successfully treated with antibiotics (Sparboe et al., 1986). In recent years, the number of outbreaks in the farmed Atlantic salmon population has substantially increased. The reasons for the most recent outbreaks remain unclear, and *Y. ruckeri* infections are nowadays a major challenge facing the Norwegian aquaculture industry, similar to other countries such as Australia (Barnes et al., 2016), Chile (Avendaño-Herrera et al., 2017), and Scotland (Ormsby et al., 2016).

Each of the human *Yersinia* pathogens harbors chromosomally and plasmid-encoded virulence determinants (Chauhan et al., 2016). *Y. pestis* usually carries two species-specific plasmids, pPCP1 and pMT1, and one highly conserved plasmid shared among the three human pathogenic *Yersiniae*, pYV (also called pCD1) (Ben-Gurion and Shafferman, 1981; Ferber and Brubaker, 1981; Haiko et al., 2009). This large 70-kb plasmid carries a type III secretion system (T3SS), Ysc. T3SS system encodes structural proteins, chaperones as well as effector proteins called Yops (*Yersinia* outer proteins) required for *Yersinia* extracellular survival. The effector proteins and the machinery for their delivery are required for infection and manipulation of host responses to overcome the action of phagocytes (Cornelis et al., 1998). Moreover, the plasmid encodes a major virulence factor, the *Yersinia* adhesin A (YadA) (Mühlenkamp et al., 2015).

Despite the economic importance, the pathogenicity of *Y. ruckeri* has not been studied in detail. Only few virulence factors are known, and to date all of these are encoded on the chromosome. These include bacterial adhesins important in establishing a successful colonization (Romalde and Toranzo, 1993). In particular, the chromosomally encoded adhesins YrInv and YrIlm might play a role in virulence (Wrobel et al., 2017). They belong to the intimin-invasin family of adhesins, which includes also InvA, the adhesin responsible for the initial bacterial attachment and colonization of host tissues in *Y. enterocolitica* and *Y. pseudotuberculosis* (Isberg and Leong, 1990; Wrobel et al., 2017). Other virulence factors described in *Y. ruckeri* include cytotoxins and haemolysins (Romalde and Toranzo, 1993), the metalloprotease Yrp1 (Secades and Guijarro, 1999), the haemolysin/cytolysin YhlA (Fernández et al., 2007), the iron uptake system ruckerbactin (Fernández et al., 2004), and a chromosomal T3SS (Liu et al., 2016). Recently, a large proteomic study of *Y. ruckeri* strains was performed under standard (Kumar et al., 2017) and iron-limited conditions (Kumar et al., 2016). In total, 1395 proteins were identified in the whole cell lysate of *Y. ruckeri* under standard culture conditions. Among them, several proteins were predicted to be virulence factors, including, among others, HtrA protease, TolB, the lipoprotein NlpD and a LuxR family transcriptional regulator. This global proteomic analysis will help in understanding the biology of the pathogen, as well as in development of new effective treatments against the ERM disease (Kumar et al., 2017).

Plasmid-borne virulence factors have been found in other fish pathogens, including *Vibrio anguillarium* (Crosa, 1980) and *Edwardsiella tarda* (Yu et al., 2012), but not in *Y. ruckeri*. Plasmids in *Y. ruckeri* strains were studied previously due to their possible involvement in virulence in analogy to the human pathogenic *Yersiniae* (De Grandis and Stevenson, 1982). Many authors expected to find the same virulence traits as those described for the human pathogens, such as the plasmid-encoded T3SS. However, none of the plasmid-associated virulence factors of the human-enteropathogenic *Yersiniae* were found in these plasmids. In general, *Y. ruckeri* plasmids have not yet been properly characterized and further research is required to understand their role in bacterial virulence. A study including 183 *Y. ruckeri* strains from different geographical locations reported 8 different plasmid profiles (Garcia et al., 1998). In this study, the most virulent sorbitol-negative *Y. ruckeri* strains of serotype O1 contained a large 75 MDa plasmid (~113 kb), in agreement with earlier studies (Guilvout et al., 1988; Romalde et al., 1993). In addition, smaller plasmids (12.7 MDa; ~19 kb) have been found in most of the strains (Garcia et al., 1998).

More recent studies showed that multidrug resistance plasmids in *Y. ruckeri* strains are a serious aquaculture concern (Toranzo et al., 1983; De Grandis and Stevenson, 1985; Carattoli et al., 2012; Huang et al., 2014). Welch et al. (2007) showed that *Y. ruckeri* strain YR71 carries a multidrug resistance plasmid called pYR1, which has 99% nucleotide identity with the IncA/C (incompatibility A/C) plasmid backbone of the *Y. pestis* isolate IP275, plasmid pIP1202. The IncA/C group comprises a large, low-copy number, multidrug resistance plasmid family within *Enterobacteriaceae* such as *Escherichia coli, Salmonella enterica, Y. pestis*, and *Klebsiella pneumoniae*, as well as more distantly related species such as *Vibrio cholerae*. Plasmids of this family are unique with regard to their structure and gene content. They contain putative transfer regions [type IV secretion system (T4SS)], regions involved in integration of mobile genetic elements, as well as regions involved in transcription (Johnson and Lang, 2012). T4SSs are widely distributed in prokaryotes as well as in some archaea. T4SSs are large macromolecular complexes typically composed of a cell-envelope spanning mating channel and an extracellular pilus structure. T4SSs are classified into two major groups type IVA (T4ASSs) and type IVB (T4BSSs). T4ASS resemble the VirB/VirD system of *Agrobacterium tumefaciens* while T4BSSs are related to the conjugation system of IncI plasmids. Typical examples of T4ASSs are found on conjugative plasmids, such as F, RP4 and pKM101, as well as the prototypical VirB system of *A. tumefaciens*. These T4ASSs export nucleoprotein complexes during conjugation. T4BSS is represented by the *Legionella pneumophila icm/dot* system involved in protein translocation into host cells thus allowing the pathogen to replicate intracellularly (Wallden et al., 2010).

In this work, we sequenced a plasmid—which we named pYR4—from the highly pathogenic Norwegian *Y. ruckeri* isolate NVH_3758 from the 1987 outbreak and performed a comparative bioinformatics analysis of the available *Y. ruckeri* plasmid sequences to evaluate their role in virulence.

## Materials and methods

### Plasmid DNA sequencing technology

Genomic DNA as well as plasmid DNA was extracted from a locally important, highly pathogenic Norwegian *Y. ruckeri* isolate NVH_3758 (biotype 1, serotype 1) recovered from an outbreak of clinical yersiniosis in farmed Atlantic salmon, kindly provided by Prof. Duncan Colquhoun at the Norwegian Veterinary Institute in Oslo, Norway (Gulla et al., 2018). Whole genome sequencing of *Y. ruckeri* NVH_3758 was performed by the Norwegian Sequencing Centre (Oslo, Norway) using the Single Molecule Real Time (SMRT) sequencing technology of Pacific Biosciences. Sample preparation, reads assembly and consensus polishing were done as previously described (Wrobel et al., 2017). The final assembly yielded two contigs of circularized length of ~3.8 Mb, representing the chromosomal genome (Wrobel et al., 2017), and ~81 kb, corresponding to a new plasmid that we named pYR4. The DNA sequence of pYR4 has been deposited in the National Centre for Biotechnology Information (NCBI) database under the accession number CP032236.

### Plasmid annotation

The FASTA consensus of pYR4 from *Y. ruckeri* NVH_3758 was uploaded to RAST (Rapid Annotation using Subsystem Technology) for automatic annotation (Aziz et al., 2008; Seemann, 2014). After initial annotations, all open reading frames (ORFs) with initial annotations were checked using the interactive server HHpred available at the Max Planck Institute for Developmental Biology Toolkit (Söding et al., 2005) against two databases, the PDB and PFAM (Sonnhammer et al., 1998; Sussman et al., 1999). The functional annotations obtained from the HHpred server and RAST were compared and in some cases were corrected manually. Many uncharacterized proteins which were previously labeled as hypothetical by RAST were annotated based on similarity to characterized proteins. The protein sequences were uploaded into Geneious (Kearse et al., 2012). A circular representation of pYR4 showing the annotated features, the GC content and the GC skew within 50 bp-long genomic regions, was generated with Circos (Krzywinski et al., 2009). The identification of the promoter sequences of the *pil* and *tra* operons was performed with the online server BPROM (Solovyev and Salamov, 2011) as well as bTSSfinder (Shahmuradov et al., 2017) (see Table 1 and Supplementary Figure 1). The mfold Web server was used for RNA secondary structure prediction (Zuker, 2003).

**Table 1:** pYR4 promoter predictions by bTSSfinder (Shahmuradov et al., 2017) and BPROM (Solovyev and Salamov, 2011) used in the present study.

**Name of operon**	**Predicted σ factor**	**Sequence of predicted promoter**		**Location on the pYR4 sequence (nt position)**	**Score^a^**	**Software used**
		**−10**	**−35**			
*pil* operon	σ^24^	TCTGT	TCATT	14,351–14,374	1.77	bTSSfinder
	σ^38^	TATTCC	TTTACC	14,339–14,368	1.52	
*tra2* operon	σ^24^	GCGAT	CCACTG	35,014–35,038	0.30	bTSSfinder
	σ^32^	CCCCCCACTG	CTCCAGA	34,989–35,019	1.91	
*mob* operon	σ^70^	TATAAT	TTGATT	60,335–60,306	1.91	bTSSfinder
	σ^38^	TATAAT	TTGATT	60,335–60,306	1.80	
	σ^32^	TACGCCAGAT	CGATTTT	60,289–60,259	1.86	
*stbA* operon	σ^32^	ATCACTATTA	TGATTGA	72,064–72,035	1.95	bTSSfinder
	σ^38^	TACACA	CGTGAG	72,030–72,002	1.94	
	σ^28^	TGAGATAA	AAAATCAA	72,011–71,939	1.97	
	σ^70^	CAATAT	TTTAAT	71,981–71,961	1.97	
	σ^24^	TCAAT	TAATAT	71,992–71,978	1.97	
*parA* operon	σ^32^	ATCACTATTA	TGATTGA	72,035–72,064	1.95	bTSSfinder
	σ^38^	TACACA	CGTGAG	72,002–72,030	1.94	
	σ^28^	TGAGATAA	AAAATCAA	71,939–72,011	1.97	
	σ^70^	CAATAT	TTTAAT	71,963–71,981	1.97	
	σ^24^	TCAAT	TAATAT	71,978–71,993	1.97	
*pil* operon	σ^70^	GCGTATTC	TTACCG	14,340–14,367	15; 55	BPROM
*tra2* operon	σ^70^	ATGAAAAAT	TTTAC	34,792–34,817	39; 42	BPROM
*tra3* operon	σ^70^	TCGCAAAAT	TTTCAG	47,970–48,003	30; 47	BPROM
*mob* operon	σ^70^	GGGTATAAT	TTGATT	60,327–60,311	53; 91	BPROM
*stbA* operon	σ^70^	CACTATTAT	TTGACA	72,065–72,038	56; 66	BPROM
*parA* operon	σ^70^	CACTATTAT	TTGACA	72,038–72,065	56; 66	BPROM

a *A score of 0.81 or higher is considered significant for bTSSfinder, and a score of 0.2 or higher is considered significant for BPROM (Solovyev and Salamov, 2011; Shahmuradov et al., 2017). Two score values provided in the table were predicted for BPROM which correspond to the Pribnow box at the −10 position and at the −35 position, respectively*.

### RepA phylogeny

The RepA protein sequence of pYR4 was annotated by RAST as “hypothetical.” After the initial annotation, the protein was identified as RepA using HHpred. The protein sequence was then subjected to a search using BLASTP (Altschul et al., 1997). The BLASTP search returned 100 hits, from which the first 29 RepA protein sequences were selected, after excluding sequences of hypothetical proteins and multispecies proteins, and aligned using MUSCLE (Edgar, 2004). In the final alignment, we included RepA protein sequences from pYR1, pYR3, pYR4 in addition to eight RepA protein sequences belonging to the IncA/C plasmid family. The final alignment was then used to construct the phylogenetic tree using MEGA X software by applying the Maximum Likelihood method on the Poisson correction model (Zuckerkandl and Pauling, 1965; Felsenstein, 1985; Kumar et al., 2018) (see Supplementary Figure 2).

### Plasmid comparative analysis

We compared the nucleotide sequence of *Y. ruckeri* NVH_3758 plasmid pYR4 with the nucleotide sequences of the plasmids of *Y. ruckeri* strains YR71 (pYR1), CSF007-82 (pYR2, pYR3) and SC09 (pLT, pWKY) (Table 2) deposited in the NCBI database. To keep the same annotation system as for the pYR4 plasmid, the nucleotide sequences of the *Y. ruckeri* plasmids were re-annotated with RAST (see also Supplementary Figure 3 for pYR4, Supplementary Figure 4). Details of the annotation can be found in Supplementary Tables 1 and 2.

**Table 2:** *Y. ruckeri* plasmid sequences deposited in GenBank used in the present study.

**Plasmid**	**Strain**	**Sequencing technology**	**Size (bp)**	**G+C content (%)**	**CDS (predicted by RAST)**	**GenBank**	**References**
pYR1	YR71	AB 3730xl	158.038	50.9	200	CP000602.1	Welch et al., 2007
pYR2	CSF007-82	PacBio	16.923	41.5	25	LN681229.1	Nelson et al., 2015
pYR3	CSF007-82	PacBio	103.917	48.4	107	LN681230.1	Nelson et al., 2015
pYR4	NVH_3758	PacBio	80.843	49.4	92	PRJNA401093	This study
pLT	SC09	Illumina	57.905	44.3	65	CP025802.1	Liu et al., 2016
pWKY	SC09	Illumina	73.051	40.2	95	CP025801.1	Liu et al., 2016

Plasmid comparisons were also done with sequences of other species containing the *tra* and *pil* operons described in literature, including *Erwina amylovora* (pEL60, pEA68, pEA72, pEA78), *Serratia entomophila* (pADAP), *Citrobacter freundii* (pCTX-M3), and *Salmonella enterica* subsp. *enterica* serovar Typhimurium (R64) (see Supplementary Figure 5). The nucleotide sequence of *Y. ruckeri* NVH_3758 pYR4 plasmid was also compared to PacBio-sequenced genomic data of *Y. ruckeri* CSF007-82, Big Creek 74, QMA0440, SC09, and Illumina-sequenced genomes of *Y. ruckeri* ATCC29473 and YRB (see Supplementary Figure 5). In the comparative survey, we also included the ~57 kb-long scaffold 20 of the *Y. ruckeri* 150 assembly, which contains the *tra* and *pil* operons.

Finally, pYR4 was compared to the Illumina-sequenced genomes of human pathogens *Y. pestis* CO92, *Y. pseudotuberculosis* YPIII, and *Y. enterocolitica* 8081 (see Supplementary Figure 5). Pairwise comparisons were performed with Progressive Mauve (Darling et al., 2010) using default options and the “seed family” option to increase sensitivity. The output backbone file was then used to plot the Locally Collinear Blocks (LCB) in a circular representation with Circos.

## Results

### pYR4 is a novel plasmid isolated from *Y. ruckeri* NHV3758

**Figure 1:**
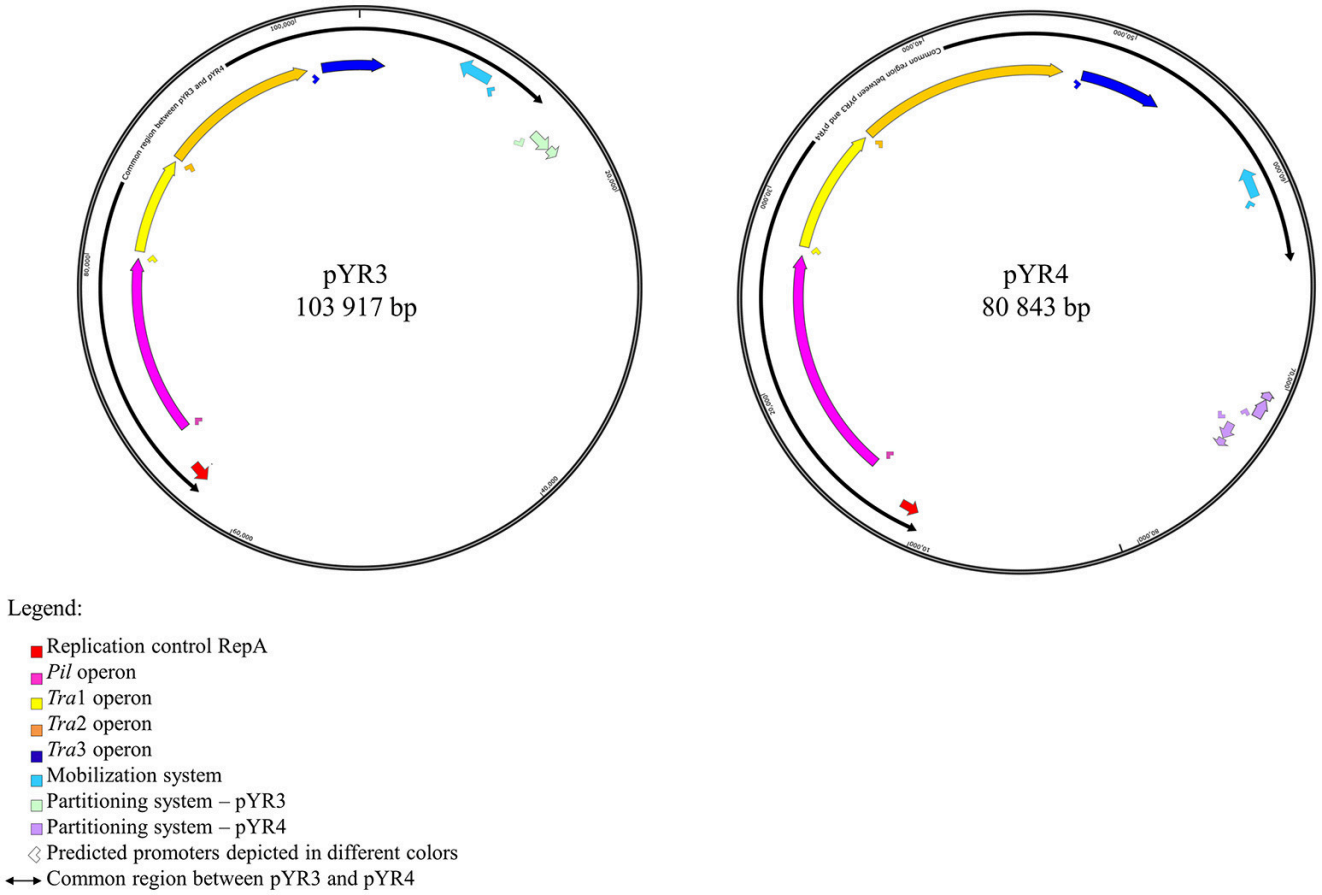
Schematic representation of plasmid maps of pYR3 and pYR4. Gene clusters are depicted in different colors: light blue (mobilization gene cluster), yellow (*tra1* gene cluster), orange (*tra2* gene cluster), dark blue (*tra3* gene cluster), magenta (*pil* gene cluster), purple (partitioning gene cluster). Approximate locations of the promoters based on the prediction of sigma factors with BPROM and bTSSfinder is reported as arrows below each operon. Replication initiation protein is indicated in red. Common regions for pYR3 and pYR4 are represented by bidirectional arrows. Partitioning system for pYR3 and pYR4 are depicted in light green and light purple, respectively. pYR4 contains 2 partitioning systems oriented in two different directions.

In order to define the relationship between the plasmid sequenced in this study and those described in literature, we performed a comparative analysis of pYR4 with plasmid sequences generated previously by Illumina and PacBio sequencing technologies. The comparative survey with Mauve indicated no obvious similarity between pYR4 and the *Y. ruckeri* plasmids pYR1, pYR2, pLT, and pWKY, as no LCBs (locally collinear blocks) were detected (data not shown). On the other hand, a ~55 kb-long LCB (sequence identity >99%) that included the *pil* and the *tra* operons was present in pYR4 (from nucleotide position 9,100 to 64,005) and in the PacBio-sequenced plasmid pYR3 (Figures 1, **3**, Table 2). By re-annotating pYR3 and comparing it with the higher-resolution annotation of pYR4 obtained through HHPred, we could provide a more in depth characterization of the plasmids under analysis (Nelson et al., 2015) (see Materials and Methods section, Supplementary Figure 3 for pYR4 and Supplementary Figure 4 for pYR3).

The remaining portion of the plasmid sequence (>25 kb, 31% of the sequence length) appears to be unique, as no LCB was found in any of the plasmids of *Y. ruckeri* deposited in GenBank so far. This region contains mostly hypothetical proteins (*n* = 10) and mobile genetic elements (*n* = 10). In addition, we found a partial toxin-antitoxin system, a restriction system including both a type I restriction enzyme and a corresponding ArdA-like anti-restriction protein, and a small cluster of genes coding for two alcohol dehydrogenase enzymes and a transcription factor with high similarity to FrmR from *Salmonella*, a formaldehyde-sensitive regulator (Supplementary Figure 3). pYR4 contains two potential partitioning systems (ParAG and StbAB) (Figure 1). These two partitioning systems are represented by two operons oriented in opposite directions. No obvious sequence similarity with the partitioning system of pYR3 was found. Interestingly, the presence of alternative partitioning systems have already been described before for pYV from *Yersinia* species (Pilla and Tang, 2018). The high abundance of mobile elements may suggest that this plasmid is likely subject to structural rearrangements and that the unique ~25 kb region of pYR4 may be the result of recent horizontal gene transfer. This is also supported by the difference in G+C content between the ~55 kb region (50.4%) and the remaining portion of the plasmid (47.1%). It is worth noting that in pYR3, this complete region is replaced by a different ~45 kb region (Figure 1). These major differences suggest that *Y. ruckeri* NHV3758 contains a plasmid with significant differences to pYR3, which we named pYR4.

### Sequence analysis of pYR4

**Figure 2:**
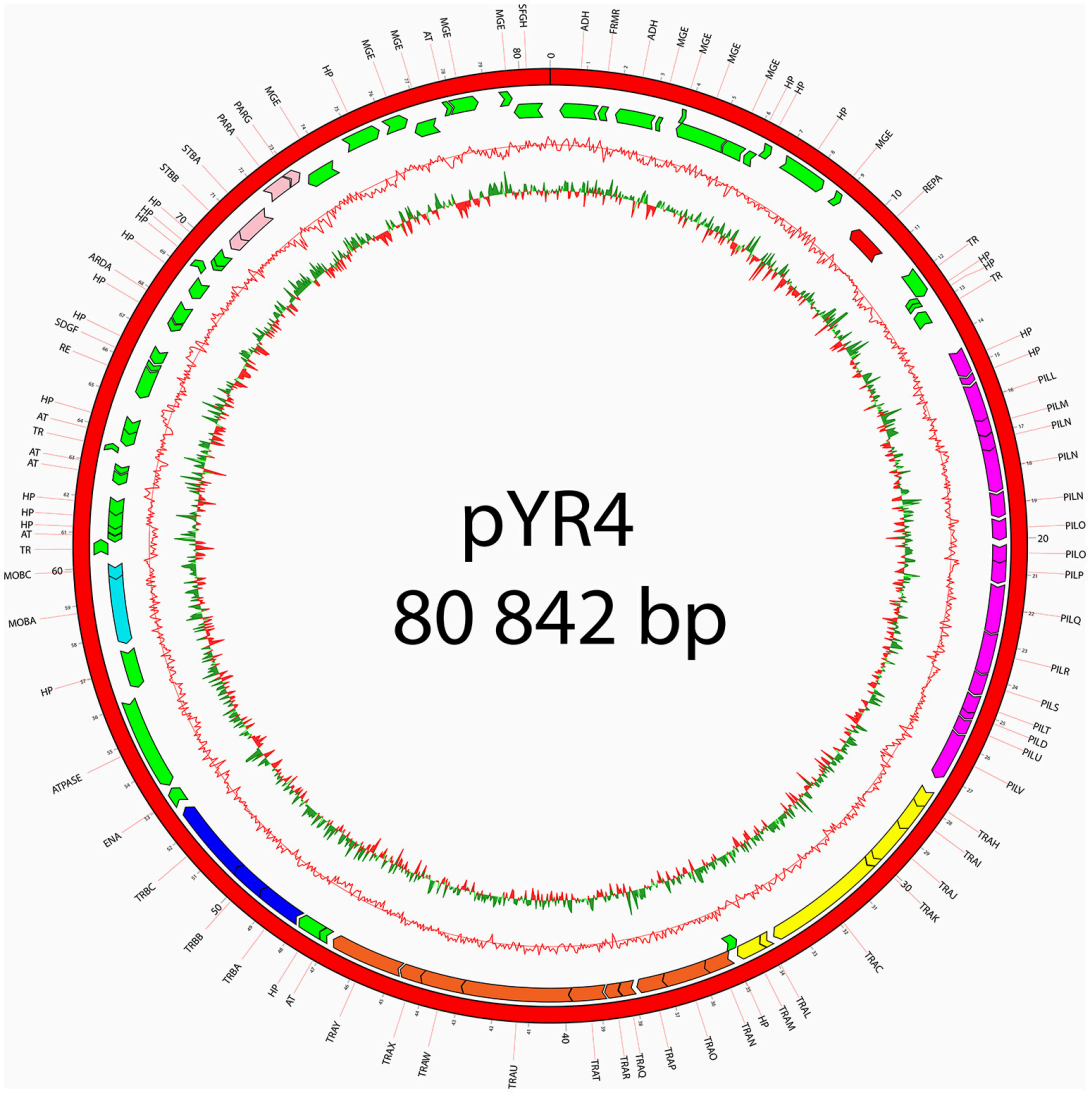
Plasmid map of pYR4 isolated from *Y. ruckeri* NVH_3758. The sequence annotations were generated with RAST (Rapid Annotation using Subsystem Technology) and further analyzed with HHpred. The rings show from inside to outside (1) the GC skew, (2) the G+C content, (3) the position of predicted ORFs in the reverse strand, and (4) the position of ORFs in the forward strand. The gene annotations are positioned in the middle of each gene on the plasmid map. Gene clusters are indicated in different colors: light blue (mobilization gene cluster), yellow (*tra1* gene cluster), orange (*tra2* gene cluster), dark blue (*tra3* gene cluster), magenta (*pil* gene cluster), pink (partitioning gene cluster). Replication initiation protein is indicated in red. Hypothetical proteins (HP) and mobile genetic elements are indicated in green.

The nucleotide sequence of the circular pYR4 plasmid contains 80,842 base pairs (~53 MDa). The G+C content of pYR4 is 49.4%, which is around 2% less than the G+C content of the *Y. ruckeri* NVH_3758 genome (47.6%), suggesting acquisition by horizontal gene transfer (Figure 2) (Nishida, 2012; Hayek, 2013). The annotation of pYR4 with RAST showed 92 putative coding sequences along the entire plasmid sequence. The RAST server could annotate functions for 52 ORFs and we were able to expand this list to 71 ORFs manually, using the HHpred server (Söding et al., 2005) (see Supplementary Figure 3), leaving 21 ORFs without putative function. Fifty-five genes are encoded on the positive strand while the remaining 37 are encoded on the negative strand. The entire plasmid sequence can be divided into several gene clusters, including clusters for partitioning (*parA, parG*, and *stbAB*), a T4SS (*tra*), and a type IV pilus (TFP) gene cluster (*pil*).

In order to understand the pYR4 plasmid physiology as well as to follow its evolution and spread, we classified pYR4 into a plasmid family. A classical method of plasmid classification is based on incompatibility (Inc) groups. In general, plasmids with the same replication system are incompatible while plasmids with different replication system are compatible. In other words, two plasmids of the same Inc group cannot be propagated in the same bacterial cell (Couturier et al., 1988). A set of 30 RepA protein sequences from IncFII plasmid family, together with 9 RepA protein sequences previously characterized as belonging to IncA/C, were aligned in order to classify pYR4. Evaluation of the RepA phylogeny showed that the pYR4 RepA protein is closely related to IncFII plasmids found in other *Yersiniae* species (see Supplementary Figure 2). A BLASTP search of pYR4 RepA protein returned over 100 hits of homologs found in different species. The closest RepA homologs were found in Illumina-sequenced genomes of *Y. frederiksenii* and *Y. enterocolitica* with 91 and 90% similarity over the whole protein sequence, respectively. These RepA homologs presumably are part of unnamed plasmids that were incorrectly assigned to chromosomes, since they share only 19% similarity to RepA of the well-described virulence plasmid pYV from *Y. enterocolitica* 8081 (Table 3). Thus, pYR4 was classified as a member of the IncFII plasmid family, in contrast to pYR1 which belongs to the IncA/C family and is represented as an outgroup in Supplementary Figure 2 (Carattoli, 2009). The IncFII plasmid family includes low-copy number plasmids mostly related to virulence, such as pYV, as well as to the dissemination of antimicrobial resistance determinants. Plasmids from this family usually carry the FII replicon alone or in association with extra replicons such as repFIA and repFIB (Carattoli, 2009; Yang et al., 2015) and are common in *Yersiniae* (Villa et al., 2010).

**Table 3:** pYR4 RepA homologs detected using BLASTP.

**Strain name**	**Name of the protein**	**Size (amino acids)**	**Degree of similarity**	**Accession number**
*Y. frederiksenii*	Replication protein	316	280/308 (91%)	WP_088130752.1
*Y. enterocolitica*	Replication protein	316	276/308 (90%)	WP_075339110.1
*Y. massiliensis*	Replication protein	316	274/308 (89%)	WP_099462805.1
*Y. kristensenii*	Replication protein	316	270/308 (88%)	WP_087768868.1
*Photorhabdus temperata* subsp. *temperata* M1021	Sea7	314	229/308 (74%)	EQB98986.1
*S. fonticola* AU-P3(3)	Sea7	362	217/308 (70%)	ERK05611.1
*S entomophila*	Sea7	362	208/306 (68%)	WP_010895766.1
*S. marcescens*	Replication protein	312	208/308 (68%)	WP_089197752.1
*S. fonticola*	Replication protein	312	204/306 (67%)	WP_074032170.1
*E. tarda*	Replication protein	311	194/303 (64%)	WP_097364799.1

### Comparative analysis of *Y. ruckeri* plasmid sequences demonstrates errors in assemblies of second generation genome sequencing

**Figure 3:**
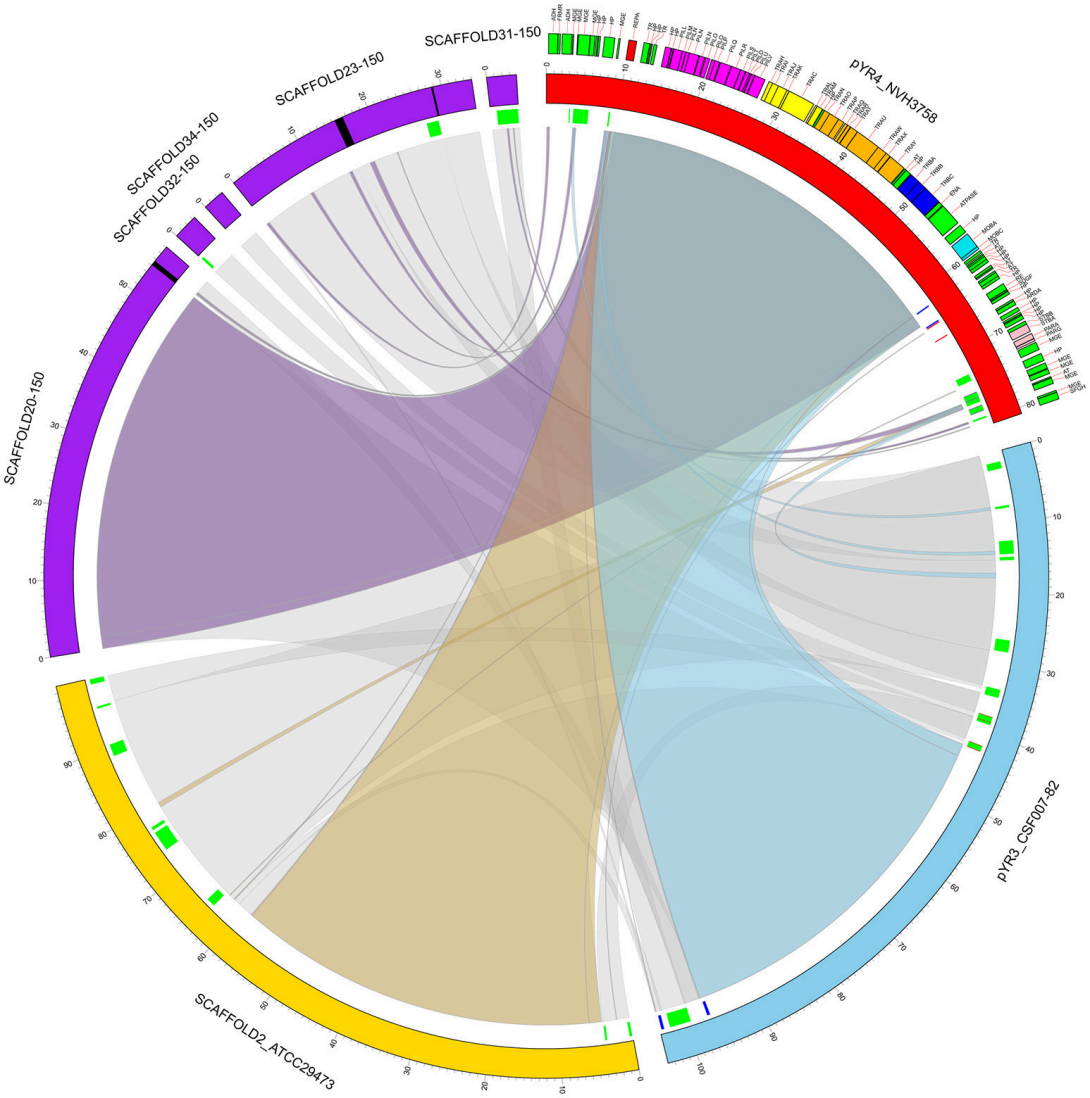
Circular representation of a comparative analysis of pYR4 from *Y. ruckeri* NVH_3758 to pYR3 and scaffolds of genomic assemblies from *Y. ruckeri* ATCC29473 (scaffold 2) and *Y. ruckeri* 150 (scaffolds 20, 23, 31, 32, 34). The pYR4 plasmid is depicted in red, pYR3 in blue, *Y. ruckeri* ATCC29473 scaffold 2 in yellow, and *Y. ruckeri* 150 scaffolds in purple. Sequence gaps (stretches of Ns) within ATCC29473 scaffolds are reported in black. Annotated features in pYR4 are shown and colored as in Figure 2. Repetitive DNA regions in pYR3 and pYR4 are colored red and blue, and mobile genetic elements are colored in green (lines in inner ring). Pairwise Locally Collinear Blocks (LCBs) as found in Mauve DNA alignments, are represented as ribbon links colored as follows: pYR4-pYR3 in light blue, pYR4-ATCC29473 in yellow, pYR4-150 in purple, pYR3-ATCC29473, and pYR3-150 in gray. Ribbon links of the ATCC29473 and 150 scaffolds with pYR3 and pYR4 are represented separately in Supplementary Figures 6, 7.

Our comparative survey showed that no significant LCBs were found between pYR4 and the chromosomal genomes of *Y. ruckeri* CSF007-82, Big Creek 74, QMA0440, SC09, or YRB (data not shown). However, we found that the ~100 kb-long scaffold 2 of the *Y. ruckeri* ATCC29473 Illumina assembly matched pYR3, except for a mobile element of pYR3 (Figure 3 and Supplementary Figures 4–6). Furthermore, the higher quality of PacBio sequenced plasmids (pYR3 of CSF007-82 and pYR4 of NHV-3758) made it possible in our comparative survey to place scaffolds of previous *Y. ruckeri* assemblies into plasmid locations. In fact, the ~57 kb-long *Y. ruckeri* 150 scaffold 20 that contained the *tra* and the *pil* operons could be mapped entirely to pYR3 (see Supplementary Figure 7). This scaffold included the ~55 kb-long region containing the *pil* and the *tra* operons detected in pYR4 and pYR3. Furthermore, by aligning other unplaced scaffolds of the *Y. ruckeri* 150 assembly, we found that four more scaffolds (23, 31, 32, 34) could be placed within pYR3 (Figure 3 and Supplementary Figures 5, 6). (An unplaced scaffold is a sequence found in an assembly that is not associated with any chromosome). Taken together, the evidence presented here suggests that the *pil* and *tra* operons are localized on plasmids pYR3 and pYR4 and that *Y. ruckeri* 150 and *Y. ruckeri* ATCC29473 contain the plasmid pYR3. In *Y. ruckeri* 150, the presence of plasmid- and chromosomally-borne *tra* clusters has been suggested based on Southern blot hybridization evidence (Méndez et al., 2009). When searching for *tra* genes in the assembly, we could not find copies of *tra* genes other than those matching pYR3 in the scaffold 20 of *Y. ruckeri* 150. Based on our data, chromosomal localization of the *tra* cluster seems very unlikely. However, resequencing or a higher quality assembly of the genome of *Y. ruckeri* 150 could clarify this unambiguously in the future.

Finally, no significant LCBs were found when comparing pYR4 or pYR3 to *Yersinia* human pathogenic species, including the well-studied pYV plasmid from *Y. enterocolitica*. This suggests a very different strategy for host infection in *Y. ruckeri*, as the pYV plasmid is essential for virulence in *Y. enterocolitica*.

### Sequence analysis of *pil* operon and its potential involvement in virulence

Analysis of the pYR4 nucleotide sequence of a putative *pil* operon showed that this region spans a 12.6 kb locus containing 17 ORFs that encode a TFP. TFPs, not to be confused with T4SSs, are surface appendages expressed by many Gram-negative bacterial species. TFPs span both bacterial membranes and they are evolutionary and structurally related to type II secretion systems. TFPs are involved in bacterial adhesion, biofilm formation, horizontal gene transfer, and pathogenesis, and in addition they mediate cell movement such as gliding motility in *Myxococcus xanthus* and twitching motility in *Pseudomonas* and *Neisseria* species (Shi and Sun, 2002). In the enteropathogenic *Y. pseudotuberculosis*, the TFP gene cluster is composed of 11 open reading frames and contributes to *Y. pseudotuberculosis* pathogenicity (Collyn et al., 2002). The arrangement of the pYR4 *pil* cluster (Figure 2; see also Table 1 and Supplementary Figure 1) resembles the *pil* cluster from the plasmid pADAP, which was described in *S. entomophila* (Hurst et al., 2011), and in plasmid R64 from *S. enterica* serovar Typhimurium (Kim and Komano, 1997). By analogy with the *pil* operon from *S. entomophila*, we adopted the same names for the putative proteins as described there, and designated them as PilLMNNNOOPQRSTDUV [(with the exception of the first two hypothetical proteins, designated as HP (H- for hypothetical and P- for proteins)]. It is worth mentioning that the *pil* operon in *S. enterica* includes only 14 genes (pi*lIJKLMNOPQRSTUV)* in contrast to pYR4 (17 genes). The number of genes for *pil* clusters can vary, as described by Zhang et al. for *S. enterica* serovar Typhi, where the *pil* operon lacks the *pilI, pilJ*, and *pilK* genes (Zhang et al., 2000) of *S. entomophila*. The overall G+C content of the *pil* cluster is 51.7%, and thus higher than that of the *Y. ruckeri* NVH_3758 genome (47.6%) and the average of pYR4 (49.4%). The G+C content of the individual genes in the *pil* locus varies between 55.1% (*pilP*) and 46.8% (*pilN*). The ORFs of the *pil* cluster are encoded on the same strand and the length of the intercistronic region between each ORF ranges from 20 to 205 bp. In the region up to 333 bp upstream of the first hypothetical protein of the *pil* cluster, we could identify binding sites for three sigma factors (σ70, σ24, and σ38) using BPROM and bTSSfinder, indicating the presence of putative promoters sequences in that region. In fact, no putative promoter sequence was identified between the *pil* genes suggesting that this region may function as an operon (Figure 2) (see Table 1 and Supplementary Figure 1). Additionally, analysis of the downstream region of *pilV* showed the presence of a palindromic sequence (5′**CTAGACAGAAT**AGCCTAGTCAATATTATCTATGGCATTAAG**ATTCTGTCAG**-3′) that could serve as a transcription terminator. The analysis of the secondary structure of this region showed a steam-loop like fold with the ΔG = −15 kcal/mol using the mfold Web server (Zuker, 2003).

The comparison of the protein sequences encoded by the *pil* operon, for example PilO and PilT, of pYR4 with database sequences using BLASTP showed from 64 to 86% identity to PilO and PilT proteins found in the IncI1 plasmid family from *Serratia* species. Accession numbers for the proteins, together with their functions, are given in Table 4.

**Table 4:** The main characteristic features of in *pil* operon identified in the pYR4 plasmid from *Y. ruckeri* NVH_3758.

**CDS**	**Homolog found by BLASTP**	**Organism**	**No. of similar amino acids/total no. of amino acids (similarity %)**	**Accession number**
PilL	Lipoprotein PilL unknown function	*Y. pseudotuberculosis*	234/356 (66%)	WP_012606417.1
PilM	Lipoprotein PilM unknown function	*S. marcescens*	109/141 (77%)	CVH07391.1
PilN	TFP formation outer membrane protein, R64 PilN family	*S. liquefaciens*	106/133 (80%)	OKP16072.1
PilN	PilN family TFP formation outer membrane protein	*S. marcescens*	338/404 (84%)	WP_089197779.1
PilN	Putative TFP operon lipoprotein	*Y. intermedia*	151/217 (70%)	CNI92757.1
PilO	IncI1 plasmid pilus assembly protein PilO	*S. fonticola*	103/160 (64%)	WP_021807907.1
PilO	IncI1 plasmid pilus assembly protein PilO	*S. fonticola*	126/153 (82%)	WP_021807907.1
PilP	TFP biogenesis protein PilP	*Serratia* sp. 14-2641	142/195 (73%)	WP_065685464.1
PilQ	IncI1 plasmid conjugative transfer ATPase PilQ	*S. fonticola*	334/424 (79%)	WP_021807909.1
PilR	General secretion pathway protein GspF	*S. marcescens*	298/380 (78%)	WP_060442021.1
PilS	Prepilin	*Serratia* sp. S4	167/197 (85%)	WP_017891024.1
PilT	Lytic transglycosylase	*Serratia* sp. S4	138/161 (86%)	WP_017891023.1
PilD	Prepilin peptidase	*S. marcescens*	22/40 (55%)	ASL85991.1
PilV	Shufflon system plasmid conjugative transfer pilus tip adhesin PilV	*Y. pekkanenii*	297/446 (67%)	CNI31753.1
PilU	Prepilin peptidase	*S. fonticola* AU-AP2C	101/135 (75%)	ERK05998.1

The biogenesis of TFPs involves a number of proteins. These are all present in the *pil* operon of pYR4, suggesting that the locus is intact and functional. The pYR4 PilS protein encodes a major pilin, which is synthesized as a prePilS. In the prePilS protein sequence we identified a hydrophilic signal peptide comprising 15 residues. A predicted cleavage site lies between the 15th (glycine) and 16th (tryptophan) residues of prePilS, which is recognized by the specific peptidase PilD (Kim and Komano, 1997). The mature PilS contains an N-terminal hydrophobic region (first 23 residues in the mature protein sequence), while the C-terminal region is rich in cysteine residues, a common feature of TFP pilins (Hurst et al., 2011). Beside the prepilins, we identified two copies of PilO and three copies of PilN, which seems to be a unique feature among plasmids from the same family. PilO and PilN are integral membrane proteins and usually exist only in one copy. An ATPase required for the assembly (PilQ) and an inner membrane protein (PilR) that we identified are also necessary for the assembly of the pili on the bacterial surface.

### Sequence analysis of the *tra* regions in pYR4

Annotations of pYR4 by HHpred (Söding et al., 2005) showed the presence of the *tra* region that we presume encodes a T4SS and is involved in conjugation. In fact, the presence of a chromosomally-borne *tra* clusters in *Y. ruckeri* 150 was previously described (Méndez et al., 2009). In our analysis, in addition to the *tra* cluster identified by Méndez et al. (*tra*1) that is probably also plasmid-borne (see above), we could identify another two *tra* clusters, which we named *tra*2 and *tra*3. The *tra*2 cluster comprises 10 genes with the gene order TraNOPQRTUWXY and an average G+C content of 53.5%, while the *tra*3 cluster is composed of 3 genes with a G+C content of 52.6%. These two *tra* clusters are preceded by two putative promoter sequences with one located upstream from the *traN* gene while another one is located upstream from the *trbA* gene. The identification of the putative promoters sequences were based on the prediction of sigma factor binding sites using BPROM and bTSSfinder (see Table 1 and Supplementary Figure 1). The presence of the two identified putative promoter sequences and the small intercistronic region between the genes suggests that these genes might function as two operons, in addition to the *tra1* operon (Figures 1, 2). The genetic organization of the *tra2* and *tra3* operons resembles the gene order of the *tra* operon found in the pADAP plasmid of *S. entomophila*, the R64 plasmid of *S. enterica* serovar *Typhimurium*, pCTX-M3 of *C. freundii*, pEL60, pEA68, pEA72, and pEA78 of *E. amylovora* (see Supplementary Figure 5), as previously suggested for the *tra1* operon (Méndez et al., 2009).

The G+C content of the *tra2* and *tra3* operons (around 53%) differs from the G+C content of the chromosomes of *Y. ruckeri* NVH_3758 (47.6%), *Y. ruckeri* Big Creek 74 (47.6%), and *Y. ruckeri* CSF007-82 (47.5%). Additionally, the *tra* region was is not present in the chromosome of the *Y. ruckeri* strains mentioned above, indicating that the *tra* region may originate from another species. Méndez et al. suggested that *S. entomophila*, the causative agent of amber disease of the New Zealand grass grub, could be the source of the *tra1* region. The G+C content of the *S. entomophila* pADAP *tra* region (*tra*1, *tra*2, *tra*3) is around 52%, which is close to the G+C content of pYR4. In addition, the gene order of that region is very similar. We suggest that the whole *tra* region encompassing *tra*1, *tra*2, and *tra*3 could have been acquired from this or a closely related *Serratia* species.

The amino acid sequences of TraH, TraI, TraJ, and TraK showed 29–34% similarity to the *L. pneumophila* T4BSS proteins such as DotD (for “defect in organelle trafficking”), DotC, DotB, and IcmT (for “intracellular multiplication”) (Wallden et al., 2010). The *icm/dot* genes are required for virulence, including intracellular growth and host cell killing (Sadosky et al., 1993; Swanson and Isberg, 1996). The pYR4 Tra proteins and their Tra homologs from *L. pneumophila* are similar in size, ranging from 87 residues (for TraK) to 385 residues (for TraJ). Interestingly, it has been shown before that a *traI* mutant strain of *Y. ruckeri* 150 was attenuated in an *in vivo* assay in rainbow trout and showed difficulty growing inside the fish (Méndez et al., 2009).

### Plasmid curing study of pYR4 in *Yersinia ruckeri* NVH_3758

In order to understand the function of pYR4 and its involvement in pathogenesis, it is desirable to obtain a plasmid-cured strain. There is a wide number of plasmid curing procedures which have been successfully used to remove plasmids in *Yersiniae*. They for example include treatments with high temperatures or introduction of an incompatible plasmid (Sheridan et al., 1998; Ni et al., 2008). The genetic stability of the pYR4 plasmid was tested by treating *Y. ruckeri* cells with high temperature. *Y. ruckeri* NVH_3758 was grown in LB medium for 10 consecutive days at 37°C with dilutions each day (Trevors, 1986). After 10 days, colony PCR was performed on 10 clones using *pil* primers that could only bind to the plasmid sequence. All tested clones were PCR-positive for the pYR4 plasmid (data not shown). Based on our high quality genomic assembly, the *pil* operon is found only on the pYR4 plasmid and is absent from the chromosome. The results obtained from this analysis indicate that pYR4 is a very stable plasmid. The conjugative ability of the plasmid may maintain it in the population and determine its stability, so that even if individual cells lose it, they get it back from their neighbors. This suggests that the plasmid cannot be cured easily in a short time frame such, as the one that we tested. As we were unable to cure pYR4 within a reasonable amount of time, this precluded performing virulence assays to check the involvement of the plasmid in pathogenesis.

## Discussion

### Higher quality of pYR4 assembly and identification of unplaced scaffolds

Comparative analysis of the *pil* region of pYR4 plasmid with other *Y. ruckeri* plasmids revealed that the *pil* region is not a common feature among *Y. ruckeri* strains. Even though we could identify different *pil* regions in some of the *Y. ruckeri* plasmids such as pWKY, we did not detect any significant sequence similarity with the *pil* region described here. Literature suggests that the *pil* operon can be encoded both on plasmids (pADAP of *S. entomophila*) (Hurst et al., 2011) and the chromosome (in *Y. pseudotuberculosis* 32777) (Collyn et al., 2002). Our data shows that in *Y. ruckeri* NVH_3758, the TFP gene cluster is plasmid-encoded and no other TFP gene cluster was detected on the chromosome. In fact, our genomic comparative survey indicates that previously deposited Illumina-sequenced genomic assemblies containing the *tra* and the *pil* operons—five scaffolds from a *Y. ruckeri* 150 assembly and a scaffold from *Y. ruckeri* ATCC29743—correspond to the plasmid pYR3. In particular for the 150 strain, failure in placing the genomic scaffolds may have occurred due to the presence of repetitive DNA sequences and of mobile elements in the flanking regions, e.g., the repetitive mobile element at positions 37,633–38,328 and 41,885–42,850 of pYR3 (Figure 3). This might also explain the presence of sequence gaps in the scaffolds 20 and 23 of ATCC29743 (Figure 3). These results show the power of the PacBio SMRT technology in producing higher-quality genomic assemblies, thanks to the longer average read lengths available.

Our study highlights the problem related to incorrect assemblies when using second generation sequencing (SGS) technologies. Short-read sequencing is often not enough to properly assemble plasmid sequences. Plasmids often contain many mobile repeat structures whose DNA length exceeds that provided by limitations of SGS technology (ranging 100–600 bp), thus generating unplaced scaffolds and mis-assemblies. The longer average read length provided by the PacBio SMRT sequencing can address some of the limitations of the SGS technologies, making it possible to correctly place genomic scaffolds even when containing repetitive regions and obtain higher quality assemblies. Sequencing the NVH_3758 genome using the PacBio technology yielded two contigs representing the chromosomal genome of ~3.8 Mb and the ~81 kb plasmid pYR4. Thanks to the PacBio platform, we could correctly determine the plasmid location of both the *tra* and the *pil* operons. The presence of the *tra* operon on the pYR4 plasmid, not on the chromosome, is reasonable as the *tra* operon encodes genes involved in bacterial conjugation and DNA transfer. We re-emphasize that these two systems, despite having similar names, are very distinct structurally and mechanistically. The T4SS is a secretion system that translocates nucleoprotein complexes or effector proteins into target cells, whereas TFP are contractile appendages mainly mediating adhesion and certain types of motility (Shi and Sun, 2002).

### TFPs in pYR4 and their potential role in virulence and conjugation

In the human pathogenic *Yersiniae*, pathogenicity is mainly related to the presence of the 70-kb virulence plasmid pYV. This plasmid encodes the Yop proteins and T3SS, which enable the bacteria to survive and multiply in the host tissues (Viboud and Bliska, 2005). Plasmids described so far in *Y. ruckeri* have recently gained more and more attention due to their potential association with virulence, although in-depth knowledge regarding their function is lacking.

Annotations of pYR4 showed 92 open reading frames. We could identify three different functional regions responsible for plasmid partitioning, a T4SS and a TFP. The present study strongly suggests that the pYR4 *pil* cluster belongs to the TFP family. In addition to attachment and motility, TFPs can be involved in DNA uptake as shown in *N. gonorrhoeae* (Wolfgang et al., 1998). Interestingly, there are examples of TFPs being involved in bacterial conjugation. For example, the PAPI-1 pathogenicity island of *P. aeruginosa* can be transferred to a recipient strain lacking this island. The mobilization of PAPI-1 was dependent on a TFP (Carter et al., 2010). The fact that the *pil* operon clusters together with the *tra* operon suggests that the two are functionally coupled and that the TFPs are involved in conjugative transfer of pYR4. However, it is also possible that the TFP is a virulence factor. We speculate that the *pil* proteins in pYR4, apart from being involved in virulence, can also be responsible for thin pilus formation required for liquid mating (Kim and Komano, 1997). Experiments carried out by Collyn et al. showed that the TFP gene cluster present in *Y. pseudotuberculosis* is not only involved in synthesis of TFPs, but also contributes to its virulence (Collyn et al., 2002). Based on these findings, we speculate that the *pil* operon present in *Y. ruckeri* could play a similarly important role in fish disease.

The difference between the G+C content of the *pil* operon (51.7%) and the average G+C content of the pYR4 plasmid (49.4%) suggests that the *pil* operon could have been acquired relatively recently by horizontal gene transfer. Likewise, the difference in G+C content compared to the *Y. ruckeri* NVH_3758 chromosome (47.6%) suggests that the plasmid has been acquired from a different species. Previous studies show that for a plasmid to be horizontally transferred, the difference in the G+C composition between the genome and the plasmid should be in the range from 1 to 5% (Hurst et al., 2011). The G+C content of the plasmid pADAP of *S. entomophila* is 53%. Most of the *pil* proteins from pYR4 (PilL, PilN, PilO, PilP, PilQ, PilR, PilS, PilT, PilD) are highly similar to *pil* proteins found in TFPs in *Serratia* species, whereas others (PilL, PilU, PilN) are more similar to those found in other *Yersiniae*. These findings, in addition to the same gene organization of the *pil* cluster, suggest that the TFP locus in pYR4 may have been acquired from *S. entomophila*, which occupies a similar aquatic environment as *Y. ruckeri* (Grimont et al., 1988).

### T4SSs and the *tra* operon

Analysis of the pYR4 protein encoding sequences allowed us to identify a complete T4SS, which we named *tra*. The corresponding coding region consists of three operons. The genetic organization of the *tra* operons (*tra*1, *tra*2, *tra*3) is very similar to that found in pADAP of *S. entomophila*, pCTX-M3 of *C. freundii* and pEL60, pEA67, pEA72, and pEA78 of *E. amylovora*, as previously described for *tra*1 (Méndez et al., 2009). Comparative analysis of the pYR4 *tra* operons to the pYR3 *tra* operons from *Y. ruckeri* CSF007-82 showed 99.9% nucleotide sequence similarity, in contrast to *tra* operons from other *Y. ruckeri* strains, where we did not detect any strong sequence similarity. In 2007, Welch et al. showed the presence of multidrug resistance of the plasmid pYR1 in *Y. ruckeri* YR71. pYR1 also contains a T4SS for conjugative transfer. However, the T4SS of pYR1 does not show high levels of sequence similarity—and no LCB was found in the Mauve alignment—to the *tra* operons described here (Welch et al., 2007).

Interestingly, similarities between the pYR4 Tra and *L. pneumophila* Icm/Dot proteins implicate a role in *Y. ruckeri* virulence. *L. pneumophila* is the causative agent of Legionnaires' disease. As an intracellular pathogen, *L. pneumophila* is able to grow and multiply within human macrophages, leading to their killing. In *L. pneumophila*, many virulence genes located in *icm* (“for intracellular multiplication”) and *dot* (“defect in organelle trafficking”) locus have been identified. Some of the Dot/Icm proteins are homologous to the Tra proteins found in plasmid R64 (Komano et al., 2000). pYR4 Tra proteins display amino acid sequence similarity to Icm/Dot proteins ranging from 29 to 34%. They are similar in size and their predicted functions are similar. However, as we could not identify any genes encoding putative effector proteins in pYR4 of NVH_3758, it is possible that the *tra* locus is purely conjugative. Nevertheless, we cannot conclusively rule out a role for the *tra* locus in virulence. Interestingly, Méndez et al. generated the *traI* mutant strain of *Y. ruckeri* 150 in which the *traI* gene was disrupted. In the *in vivo* competition assays in rainbow trout, the virulence of the *traI* mutant strain was reported to be attenuated when compared to the WT strain, suggesting that TraI is involved in virulence of this bacterium.

## Conclusions

In conclusion, the results presented here suggest that pYR4 from the 1987 outbreak strain *Y. ruckeri* NVH_3758 is a conjugative plasmid that encodes a T4SS and a TFP that might contribute to *Y. ruckeri* virulence. The 55 kDa plasmid backbone is identical to that of pYR3 and has presumably been acquired by horizontal gene transfer through conjugation from a *Serratia* species that occupies the same biological niche, as previously suggested (Méndez et al., 2009). In addition, pYR4 contains a a previously undescribed ~25 kDa region with a partitioning system completely different from that of pYR3. This region contains a set of mobile elements, several hydrogenase enzymes with a corresponding transcription factor, and additional hypothetical genes that need further investigation.

The plasmid could contribute to the dissemination of *Y. ruckeri* virulence by spreading the TFP-encoding *pil* locus among non-virulent strains. Further experiments are required to elucidate the function of the *pil* and *tra* regions and to clarify their role in virulence. However, we would like to highlight that based on 100% sequence similarity between TraI of pYR4 and TraI of *Y. ruckeri* 150 that we now know is located on pYR3 based on our re-assembly of the *Y. ruckeri* 150 genome, we assume that at least the *tra* region is directly involved in virulence. *traI* deletion in *Y. ruckeri* 150 lead to a significant decrease in virulence (Méndez et al., 2009). Additionally, our study demonstrates the power of PacBio SMRT sequencing technology in producing assemblies of high quality and accuracy, compared to sequencing technologies based on shorter read lengths such as Illumina and 454. The latter methods failed to show the plasmid localization of the *pil* and *tra* regions in multiple cases.

## Data availability

The sequence of pYR4 described herein has been deposited in GenBank with the accession number CP032236.

## Author contributions

AW and CO conceived, planned, and carried out the experiments. AW, CO, JL, and DL contributed to the discussion and interpretation of the results, and wrote the manuscript. All authors provided critical feedback and contributed to the final shape of the manuscript.

### Conflict of interest statement

The authors declare that the research was conducted in the absence of any commercial or financial relationships that could be construed as a potential conflict of interest. The reviewer HL and handling Editor declared their shared affiliation.
