# Supplementary material for: pYR4 From a Norwegian Isolate of Yersinia ruckeri Is a Putative Virulence Plasmid Encoding Both a Type IV Pilus and a Type IV Secretion System
Source: Front Cell Infect Microbiol. 2018 Oct 30;8:373. doi: 10.3389/fcimb.2018.00373 (PMC6232867; doi:10.3389/fcimb.2018.00373)
Supplement: Supplementary file 3 [file Data_Sheet_1.pdf]

frontiers

Supplementary Material

pYR4 from a Norwegian isolate of *Yersinia ruckeri* is a putative virulence plasmid encoding a type IV pilus

Agnieszka Wrobel*, Claudio Ottoni, Jack C. Leo, Dirk Linke

***Correspondence:** Dirk Linke: dirk.linke@ibv.uio.no

Supplementary figure 1.

Putative promoters prediction for pYR4 operons using bTSSfinder (1) (Shahmuradov *et al.*, 2017) and BPROM (2) (Solovyev and Salamov, 2011) using default parameters. Raw data is included. bTSSfinder predicts putative promoters for five classes of σ factors in Cyanobacteria (σ^A, σ^C, σ^H, σ^G and σ^F) and for five classes of sigma factors in *E. coli* (σ^{70}, σ^{38}, σ^{32}, σ^{28} and σ^{24}) while BPROM predicts putative promoters for one class of σ factors, namely σ^{70}.

1) Putative promoters prediction using bTSSfinder (Shahmuradov *et al.*, 2017)

a) Putative promoter prediction upstream from hypothetical protein (*pil* operon)
Input sequence:
CTTGTCTTCGAACCTAGTTTGCTGGGGTTCTAATTACACTCCCGGCATTTAGCAGTTAACCCGGCCTTGTGTC
GGGCTCTTTTCCCCCAGATGCGTTACTACAACACTGGATGCCGGCAATGGTGTCTGAGCTGGTCCTGCCATG
GACATCCCTATCCCTTTACCGGCTGGTCATTTATGGCGTATTCCTTCTGTTGGAGGGTGATACAGACACCAT
TGCCGGCATTCGATACACCAACTCAATTAAAAGGAGATAGCCT

Search for sigma 70, 24, 32, 28 and 38 promoters
Input sequence file with query sequence(s): /var/www/btssfinder/cgi-bin/userdata/928.fasta
Search parameters: for *E. coli*
TSSs of different Sigma classes are processed separately
Search only on Positive Strand
Total scoring threshold for
sigma70: 0.06
sigma24: 0.00
sigma32: 1.01
sigma28: 1.24
sigma38: 0.31
Length of Query sequence: 260
sigma24(+): 202 Score 1.77 -35 (172) tcattt >>> tctgt -10 (195)
sigma38(+): 201 Score 1.52 -35 (160) tttacc >>> tattcc -10 (189)
Total: 2 promoter(s) predicted

b) No putative promoters were found upstream from *pilL*
Input sequence:
CTCCTCTCGTCACCCTAGCAATATCGTCCCCATATACATCAATCGATATGGCTATTCCATTACCTGATGAAA
CCTAT

c) Putative promoter prediction upstream from *traN*
Input sequence:
TCAATCATCACCCGCGATGCCCGCTAATTTTACTCAGGTACTCTCATGAAAAATCTCACTCTCACTGCAGTG
GTTATGCTGCTGTCAGCAGTCCCGGCACTCGCGACAGATTGGGTACCAGCCCGCACGGGTAATACACCAAC

CACCGAGATCCCAGCGGCACCGAAACCTACAGCACCTCAAAACGAACCTGTAGTCCCTCAAACAGATATCA
CTGCCCTGATGCTCCAGACACCACCGCCCACCCCCCCCACTGCAGCCCAAGCATGGGCGATGAGCCAGGCC
AACCCTCTGGATAATCGCCAGGGTGTTGAAATGGTAGGCAAAGTGATG

Search for sigma 70, 24, 32, 28 and 38 promoters
Input sequence file with query sequence(s): /var/www/btssfinder/cgi-bin/userdata/812.fasta
Search parameters: for *E. coli*
TSSs of different Sigma classes are processed separately
Search on Positive Strand
Total scoring threshold for
sigma70: 0.06
sigma24: 0.00
sigma32: 1.01
sigma28: 1.24
sigma38: 0.31
Length of Query sequence: 333
sigma24(+): 282 Score 0.30 -35 (251) ccactg >>> gcgat -10 (275)
sigma32(+): 261 Score 1.91 -35 (226) ctccaga >>> ccccccactg -10 (256)
Total: 2 promoter(s) predicted

d) No putative promoters were found upstream of *trbA*
Input sequence:
ACGTATCGCCGACGAGCAGGGGCTTAACCTGATCCTGCCGCCAAACTAATTCCCCTTCTGCAGTAACCCAC
AGTACCGACTCTGGGGCACCCTTTTCAGGGTGCCTCTTCTTTTGCATTCGCAAAATATTTATAGGTCTCCTCT
ATGGCGCAACCCATTCGCAAT

e) No putative promoters were found upstream of *ena*
Input sequence:
GATGATTTTGATAACACAACCGAACATCTTACCTACTGAAATAAATACTTCCCCGCCAGCTCCTCCAACCGT
TGACTAAACCATTAGCTCAATAAGCTCAATTTTTTAATCATTAAGGAATGAAAATGACTATCCGTACATTAC
GGTCAATTACTTTGGCCGGCGTTCTAC

f) Putative promoter prediction upstream of *mobC*
Input sequence:
TGGCGTTTTTCACTCTTGCTCATCTTCAATCCTGCTTAACCGCTGAGGCGGACACTGCAAGTAGGGTGTCGG
GACGGAGTCCTGACCAGGTCGTTTTGTGGGACCGACTGCGTGCAGGCGGTGCTACAAAACATACCCTGTCC
CACGTTTTACTTCAGTATTCGCTATTATGGCCGATTTTCTTTATAAAGTCATTACGCCAGATGTGGAATGGG
GTACCTTTGATTCATGAGTGAATACAAGGGTATAATTGGAAAGTTAACTCTGCACAAAGCACAGAAGGATA
TGAGGATGGCCGCAAACAGT

Search for sigma 70, 24, 32, 28 and 38 promoters
Input sequence file with query sequence(s): /var/www/btssfinder/cgi-bin/userdata/820.fasta
Search parameters: for *E. coli*
TSSs of different Sigma classes are processed separately
Search on Positive Strand
Total scoring threshold for
sigma70: 0.06
sigma24: 0.00
sigma32: 1.01
sigma28: 1.24
sigma38: 0.31
Length of Query sequence: 306
sigma70(+): 256 Score 1.91 -35 (222) ttgatt >>> tataat -10 (251)
sigma38(+): 255 Score 1.80 -35 (222) ttgatt >>> tataat -10 (251)
sigma32(+): 210 Score 1.86 -35 (175) cgattt >>> tacgccagat -10 (205)

Total: 3 promoter(s) predicted

g) Putative promoter prediction upstream of *stbA*
Input sequence:
CAAAATACATTCATCATTTTGAACCTCATCAATACTCAAACTGAGTGGTTGAAGTGCACCCAAGAAATGCA
GCTCACAATTTAGGCGATTCCCCATAATCTATGTCCGGTCAGTTACTCAAATTGGGGTGTAATCATATTAAT
ATCATTACGCAATCAAAATCAACCTAAAATGATATTGTTTTAATATTAAAACAATATTAAAAAATCAATATT
CAACCCGTGAGATAAAAGAGAAGGTGCGTACACAGTAATGATTGACAATCAATAGTTAATCACTATTATCT
TAATATTATTTTAATATTAAAAGGACTAACTATGATACTTACAGTTGGTAATACAA

Search for sigma 70, 24, 32, 28 and 38 promoters
Input sequence file with query sequence(s): /var/www/btssfinder/cgi-bin/userdata/826.fasta
Search parameters: for *E. coli*
TSSs of different Sigma classes are processed separately
Search on Positive Strand
Total scoring threshold for
sigma70: 0.06
sigma24: 0.00
sigma32: 1.01
sigma28: 1.24
sigma38: 0.31
Length of Query sequence: 342
sigma32(+): 288 Score 1.95 -35 (254) tgattga >>> atcactatta -10 (283)
sigma38(+): 257 Score 1.94 -35 (221) cgtgag >>> tacaca -10 (249)
sigma28(+): 237 Score 1.97 -35 (204) aaaatcaa >>> tgagataa -10 (230)
sigma70(+): 221 Score 1.97 -35 (182) tttaat >>> caatat -10 (214)
sigma24(+): 219 Score 1.97 -35 (184) taatat >>> tcaat -10 (212)

Total: 5 promoter(s) predicted

h) Putative promoter prediction upstream of *parA*
Input sequence:
CAAAATACATTCATCATTTTGAACCTCATCAATACTCAAACTGAGTGGTTGAAGTGCACCCAAGAAATGCA
GCTCACAATTTAGGCGATTCCCCATAATCTATGTCCGGTCAGTTACTCAAATTGGGGTGTAATCATATTAAT
ATCATTACGCAATCAAAATCAACCTAAAATGATATTGTTTTAATATTAAAACAATATTAAAAAATCAATATT
CAACCCGTGAGATAAAAGAGAAGGTGCGTACACAGTAATGATTGACAATCAATAGTTAATCACTATTATCT
TAATATTATTTTAATATTAAAAGGACTAACTATGATACTTACAGTTGGTAATACAA

Search for sigma 70, 24, 32, 28 and 38 promoters
Input sequence file with query sequence(s): /var/www/btssfinder/cgi-bin/userdata/823.fasta
Search parameters: for *E. coli*
TSSs of different Sigma classes are processed separately
Search on Positive Strand
Total scoring threshold for
sigma70: 0.06
sigma24: 0.00
sigma32: 1.01
sigma28: 1.24
sigma38: 0.31
Length of Query sequence: 342
sigma32(+): 288 Score 1.95 -35 (254) tgattga >>> atcactatta -10 (283)
sigma38(+): 257 Score 1.94 -35 (221) cgtgag >>> tacaca -10 (249)
sigma28(+): 237 Score 1.97 -35 (204) aaaatcaa >>> tgagataa -10 (230)
sigma70(+): 221 Score 1.97 -35 (182) tttaat >>> caatat -10 (214)
sigma24(+): 219 Score 1.97 -35 (184) taatat >>> tcaat -10 (212)

Total: 5 promoter(s) predicted

2) Putative promoters prediction using BPROM (Solovyev and Salamov, 2011)

a) Putative promoter prediction upstream from hypothetical protein (*pil* operon)
Input sequence:
CTTGTCTTCGAACCTAGTTTGCTGGGGTTCTAATTACACTCCCGGCATTTAGCAGTTAACCCGGCCTTGTGTC
GGGCTCTTTTCCCCCAGATGCGTTACTACAACACTGGATGCCGGCAATGGTGTCTGAGCTGGTCCTGCCATG
GACATCCCTATCCCTTTACCGGCTGGTCATTTATGGCGTATTCCTTCTGTTGGAGGGTGATACAGACACCAT
TGCCGGCATTCGATACACCAACTCAATTAAAAGGAGATAGCCT

Length of sequence- 260
Threshold for promoters - 0.20
Number of predicted promoters - 1
Promoter Pos: 196 LDF- 1.43
-10 box at pos. 181 GCGTATTC Score 15
-35 box at pos. 16 TTACCG Score 32
Oligonucleotides from known TF binding sites:
For promoter at 196:
rpoS17: GGAGGGTG at position 197 Score - 12

b) No putative promoters were found upstream from *pilL*
Input sequence:
CTCCTCTCGTCACCCTAGCAATATCGTCCCCATATACATCAATCGATATGGCTATTCCATTACCTGATGAAA
CCTAT

c) Putative promoter prediction upstream from *traN*
Input sequence:
TCAATCATCACCCGCGATGCCCGCTAATTTTACTCAGGTACTCTCATGAAAAATCTCACTCTCACTGCAGTG
GTTATGCTGCTGTCAGCAGTCCCGGCACTCGCGACAGATTGGGTACCAGCCCGCACGGGTAATACACCAAC
CACCGAGATCCCAGCGGCACCGAAACCTACAGCACCTCAAAACGAACCTGTAGTCCCTCAAACAGATATCA
CTGCCCTGATGCTCCAGACACCACCGCCCACCCCCCCCACTGCAGCCCAAGCATGGGCGATGAGCCAGGCC
AACCCTCTGGATAATCGCCAGGGTGTTGAAATGGTAGGCAAAGTGATG

Length of sequence- 333
Threshold for promoters - 0.20
Number of predicted promoters - 1
Promoter Pos: 61 LDF- 1.52
-10 box at pos. 46 ATGAAAAAT Score 39
-35 box at pos. 29 TTTACT Score 42
Oligonucleotides from known TF binding sites:
No such sites for promoter at 61

d) Putative promoter predicted upstream of *trbA*
Input sequence:
ACGTATCGCCGACGAGCAGGGGCTTAACCTGATCCTGCCGCCAAACTAATTCCCCTTCTGCAGTAACCCAC
AGTACCGACTCTGGGGCACCCTTTTCAGGGTGCCTCTTCTTTTGCATTCGCAAAATATTTATAGGTCTCCTCT
ATGGCGCAACCCATTCGCAAT

Length of sequence- 165
Threshold for promoters - 0.20
Number of predicted promoters - 1
Promoter Pos: 134 LDF- 1.65
-10 box at pos. 119 TCGCAAAAT Score 47
-35 box at pos. 94 TTTCAG Score 30
Oligonucleotides from known TF binding sites:
No such sites for promoter at 134

e) Predicted putative promoters were found downstream of *trbC*
Input sequence:
AACACAACCGAACATCTTACCTACTGAAATAAATACTTCCCCGCCAGCTCCTCCAACCGTTGACTAAACCA
TTAGCTCAATAAGCTCAATTTTTAATCATTAAGGAATGAAAATGACTATCCGTACATTACGGTCAATTACT
TTGGCCGGCGTTCTACTTTTGGCTGTCAGTTCTGCCGTTGCAGCCCCGACG

Length of sequence- 194
Threshold for promoters - 0.20
Number of predicted promoters - 1
Promoter Pos: 93 LDF- 2.55
-10 box at pos. 78 CAATAAGCT Score 31
-35 box at pos. 60 TTGACT Score 61
Oligonucleotides from known TF binding sites:
For promoter at 93:
rpoD17: CTAAACCA at position 64 Score - 6

f) Putative promoter prediction upstream of *mobC*
Input sequence:
TGGCGTTTTTCACTCTTGCTCATCTTCAATCCTGCTTAACCGCTGAGGCGGACACTGCAAGTAGGGTGTCGG
GACGGAGTCCTGACCAGGTCGTTTTGTGGGACCGACTGCGTGCAGGCGGTGCTACAAAACATACCCTGTCC
CACGTTTTACTTCAGTATTCGCTATTATGGCCGATTTTCTTTATAAAGTCATTACGCCAGATGTGGAATGGG
GTACCTTTGATTCATGAGTGAATACAAGGGTATAATTGGAAAGTTAACTCTGCACAAAGCACAGAAGGATA
TGAGGATGGCCGCAAACAGT

Length of sequence- 306
Threshold for promoters - 0.20
Number of predicted promoters - 1
Promoter Pos: 258 LDF- 5.32
-10 box at pos. 243 GGGTATAAT Score 91
-35 box at pos. 222 TTGATT Score 53
Oligonucleotides from known TF binding sites:
No such sites for promoter at 258

g) Putative promoter prediction downstream of *stbB*
Input sequence:
AGAATTATTTGAGTAAGTTTGTGCATGAGAGTTACTCCAGTTATTCGCGGCACCATTGCCGTGCCGCCACGA
CAGTCCTGCGGATAGTGGAGAAAACAAGGGCAGCGCCGCAAGCCGCAGCGCAGGCGTAAACGGGCTGAAT
GCCCTGTTTACGGTGAGAACGGGTGAGGACGTTGGCGTCAGCGTTCCCCTTGTTTGTGAAACGGCAGGGCT
ACAGTGAAGGCACGGCAAAAGGGGATATTCATACGTGAATAAATAGAACTCAGTTATCACCTAAAATTGT
AGATGACTTTTCACTCCCCTTCCCTACCGGATTTTGGCATGAGCTGTCAGGGCTAACGTATTGAGTAAGCA
GCAAACACATTACATGAAGCCCGACATTTTCTT

Length of sequence- 388
Threshold for promoters - 0.20
Number of predicted promoters - 1
Promoter Pos: 287 LDF- 5.40
-10 box at pos. 272 ACCTAAAAT Score 62
-35 box at pos. 248 GTGAAT Score 22
Oligonucleotides from known TF binding sites:
For promoter at 287:
argR: ATATTCAT at position 238 Score - 7
rpoD17: AATAAATA at position 251 Score - 11
cytR: ATAGAACT at position 256 Score - 7
rpoD19: CACCTAAA at position 271 Score - 6
rpoN: AAATTGTA at position 277 Score - 8

h) Putative promoter prediction upstream of *stbA*
Input sequence:

CAAAATACATTCATCATTTTGAACCTCATCAATACTCAAACTGAGTGGTTGAAGTGCACCCAAGAAATGCA
GCTCACAATTTAGGCGATTCCCCATAATCTATGTCCGGTCAGTTACTCAAATTGGGGTGTAATCATATTAAT
ATCATTACGCAATCAAAATCAACCTAAAATGATATTGTTTTAATATTAAAACAATATTAAAAAATCAATATT
CAACCCGTGAGATAAAAGAGAAGGTGCGTACACAGTAATGATTGACAATCAATAGTTAATCACTATTATCT
TAATATTATTTTAATATTAAAAGGACTAACTATGATACTTACAGTTGGTAATACAA

Length of sequence- 342
Threshold for promoters - 0.20
Number of predicted promoters - 1
Promoter Pos: 292 LDF- 5.33
-10 box at pos. 276 CACTATTAT Score 56
-35 box at pos. 257 TTGACA Score 66
Oligonucleotides from known TF binding sites:
For promoter at 292:
lrp: TTGACAAT at position 257 Score - 7
rpoD17: AATAGTTA at position 266 Score - 13

i) Putative promoter prediction upstream of *parA*
Input sequence:
CAAAATACATTCATCATTTTGAACCTCATCAATACTCAAACTGAGTGGTTGAAGTGCACCCAAGAAATGCA
GCTCACAATTTAGGCGATTCCCCATAATCTATGTCCGGTCAGTTACTCAAATTGGGGTGTAATCATATTAAT
ATCATTACGCAATCAAAATCAACCTAAAATGATATTGTTTTAATATTAAAACAATATTAAAAAATCAATATT
CAACCCGTGAGATAAAAGAGAAGGTGCGTACACAGTAATGATTGACAATCAATAGTTAATCACTATTATCT
TAATATTATTTTAATATTAAAAGGACTAACTATGATACTTACAGTTGGTAATACAA

Length of sequence- 342
Threshold for promoters - 0.20
Number of predicted promoters - 1
Promoter Pos: 292 LDF- 5.33
-10 box at pos. 276 CACTATTAT Score 56
-35 box at pos. 257 TTGACA Score 66
Oligonucleotides from known TF binding sites:
For promoter at 292:
lrp: TTGACAAT at position 257 Score - 7
rpoD17: AATAGTTA at position 266 Score - 13

Supplementary figure 2.

Molecular Phylogenetic analysis of pYR4 RepA protein to its closest homologs from different species within IncFII and IncA/C plasmid family. The tree shows two different clusters which resemble two different plasmid groups including IncFII (depicted in green) and IncA/C plasmid family (depicted in red). Note that the pYR4 RepA falls into the same cluster as other RepA *Yersiniae* protein sequences from the same plasmid family. The tree with the highest log likelihood (-5236.58) is shown. The percentage of trees in which the associated taxa clustered together is shown next to the branches. Initial tree(s) for the heuristic search were obtained automatically by applying Neighbor-Join and BioNJ algorithms to a matrix of pairwise distances estimated using a JTT model, and then selecting the topology with superior log likelihood value. The tree is drawn to scale, with branch lengths measured in the number of substitutions per site. The analysis involved 40 amino acid sequences. All positions containing gaps and missing data were eliminated. There were a total of 210 positions in the final dataset. Evolutionary analyses were conducted in MEGA X.

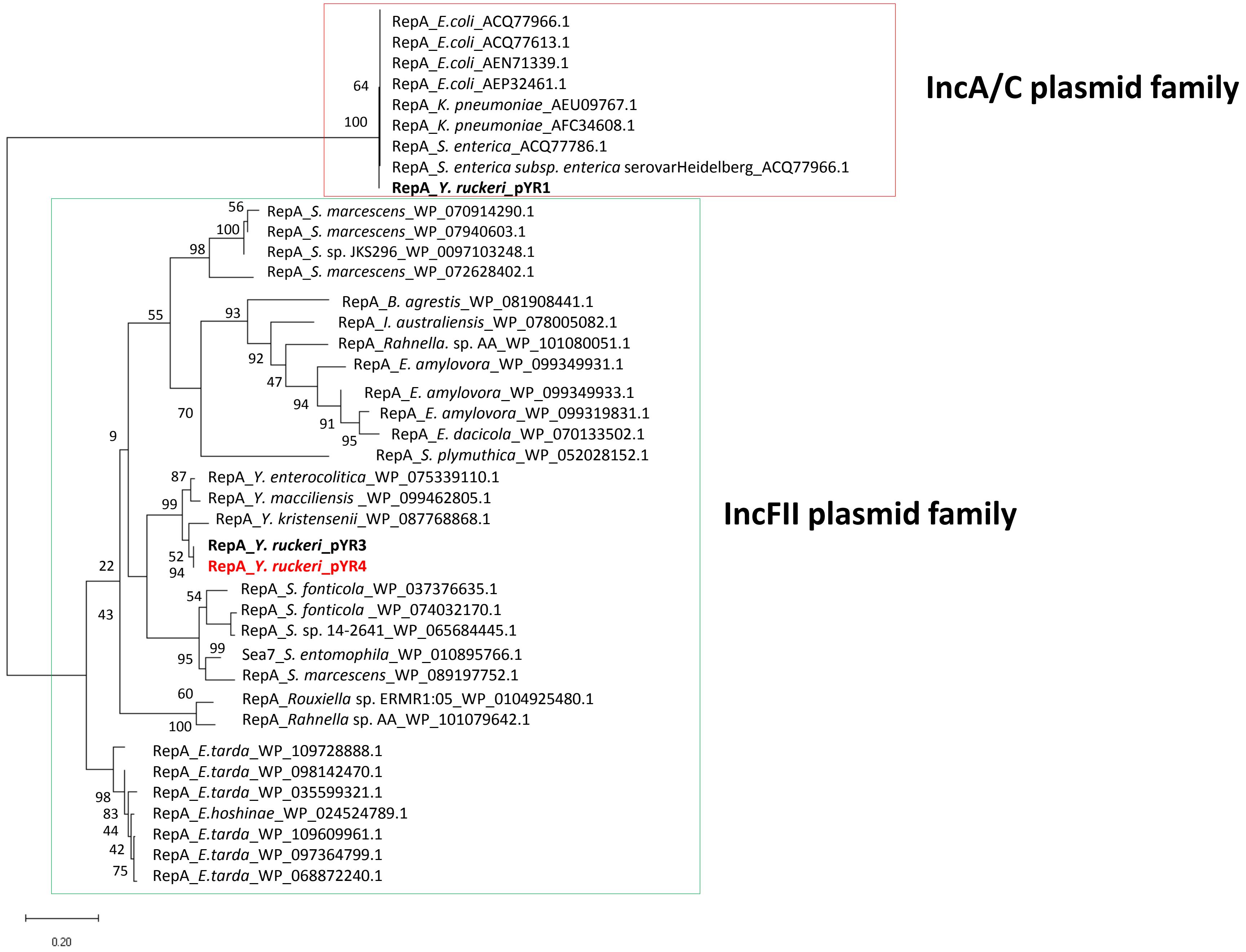

IncA/C plasmid family
IncFII plasmid family
RepA_E.coli_ACQ77966.1
RepA_E.coli_ACQ77613.1
RepA_E.coli_AEN71339.1
RepA_E.coli_AEP32461.1
RepA_K. pneumoniae_AEU09767.1
RepA_K. pneumoniae_AFC34608.1
RepA_S. enterica_ACQ77786.1
RepA_S. enterica subsp. enterica serovarHeidelberg_ACQ77966.1
RepA_Y. ruckeri_pYR1
RepA_S. marcescens_WP_070914290.1
RepA_S. marcescens_WP_07940603.1
RepA_S. sp. JKS296_WP_0097103248.1
RepA_S. marcescens_WP_072628402.1
RepA_B. agrestis_WP_081908441.1
RepA_I. australiensis_WP_078005082.1
RepA_Rahnella. sp. AA_WP_101080051.1
RepA_E. amylovora_WP_099349931.1
RepA_E. amylovora_WP_099349933.1
RepA_E. amylovora_WP_099319831.1
RepA_E. dacicola_WP_070133502.1
RepA_S. plymuthica_WP_052028152.1
RepA_Y. enterocolitica_WP_075339110.1
RepA_Y. macciliensis _WP_099462805.1
RepA_Y. kristensenii_WP_087768868.1
RepA_Y. ruckeri_pYR3
RepA_Y. ruckeri_pYR4
RepA_S. fonticola_WP_037376635.1
RepA_S. fonticola _WP_074032170.1
RepA_S. sp. 14-2641_WP_065684445.1
Sea7_S. entomophila_WP_010895766.1
RepA_S. marcescens_WP_089197752.1
RepA_Rouxiella sp. ERMR1:05_WP_0104925480.1
RepA_Rahnella sp. AA_WP_101079642.1
RepA_E.tarda_WP_109728888.1
RepA_E.tarda_WP_098142470.1
RepA_E.tarda_WP_035599321.1
RepA_E.hoshinae_WP_024524789.1
RepA_E.tarda_WP_109609961.1
RepA_E.tarda_WP_097364799.1
RepA_E.tarda_WP_068872240.1
64
100
56
100
98
93
55
92
47
94
91
95
70
9
87
99
52
94
22
54
43
99
95
60
100
98
83
44
42
75
0.20

Supplementary figure 3.

pYR4 plasmid annotations obtained by employing RAST (Rapid Annotation using Subsystem Technology)(Aziz *et al.*, 2008) and HHpred available at the Max Planck Institute for Developmental Biology Toolkit (Söding *et al*., 2005)

Supplementary figure 4.

Y. ruckeri plasmids annotations obtained using RAST (Rapid Annotation using Subsystem Technology)(Aziz *et al*., 2008).The file contains 6 different *Y. ruckeri* plasmid sequences (nucleotide and protein) including pYR1, pYR2, pYR3, pYR4, pLT, pWKY together with their proposed functions.

Supplementary figure 5.

Gen Bank Accession numbers for genome sequences (plasmid/chromosome) used in the study.

**Species**	**Genomic information**	**GenBank**
E. amylovora	pEL60	AY422214
	pEA68	HG813238
	pEA72	NC_013973
	pEA78	NC_023072
S. entomophila	pADAP	AF135182
C. freundii	pCTX-M3	AF550415
S. enterica subsp. *enterica* serovar Typhimurium	R64	NC_005014.1
Y. ruckeri CSF007-82	Chromosome	LN681231
Y. ruckeri Big Creek 74	Chromosome	NZ_CP011078.1
Y. ruckeri QMA0440	Chromosome	CP017236.1

Y. ruckeri SC09	Chromosome	CP025800.1
Y. ruckeri ATCC29473	Chromosome	KN150747.1<br>KN150748.1
Y. ruckeri YRB	Chromosome	CP009539.1
Y. ruckeri 150	Chromosome	GCF_001750505.1
Y. pestis CO92	Chromosome	AL590842
Y. pseudotuberculosis YPIII	Chromosome	CP000950.1
Y. enterocolitica 8081	Chromosome	NC_008800.1

Supplementary figure 6.

Circular representation of comparative analysis as in Figure 2 showing ribbon links of ATCC29473 with the two plasmids, pYR3 and pYR4.

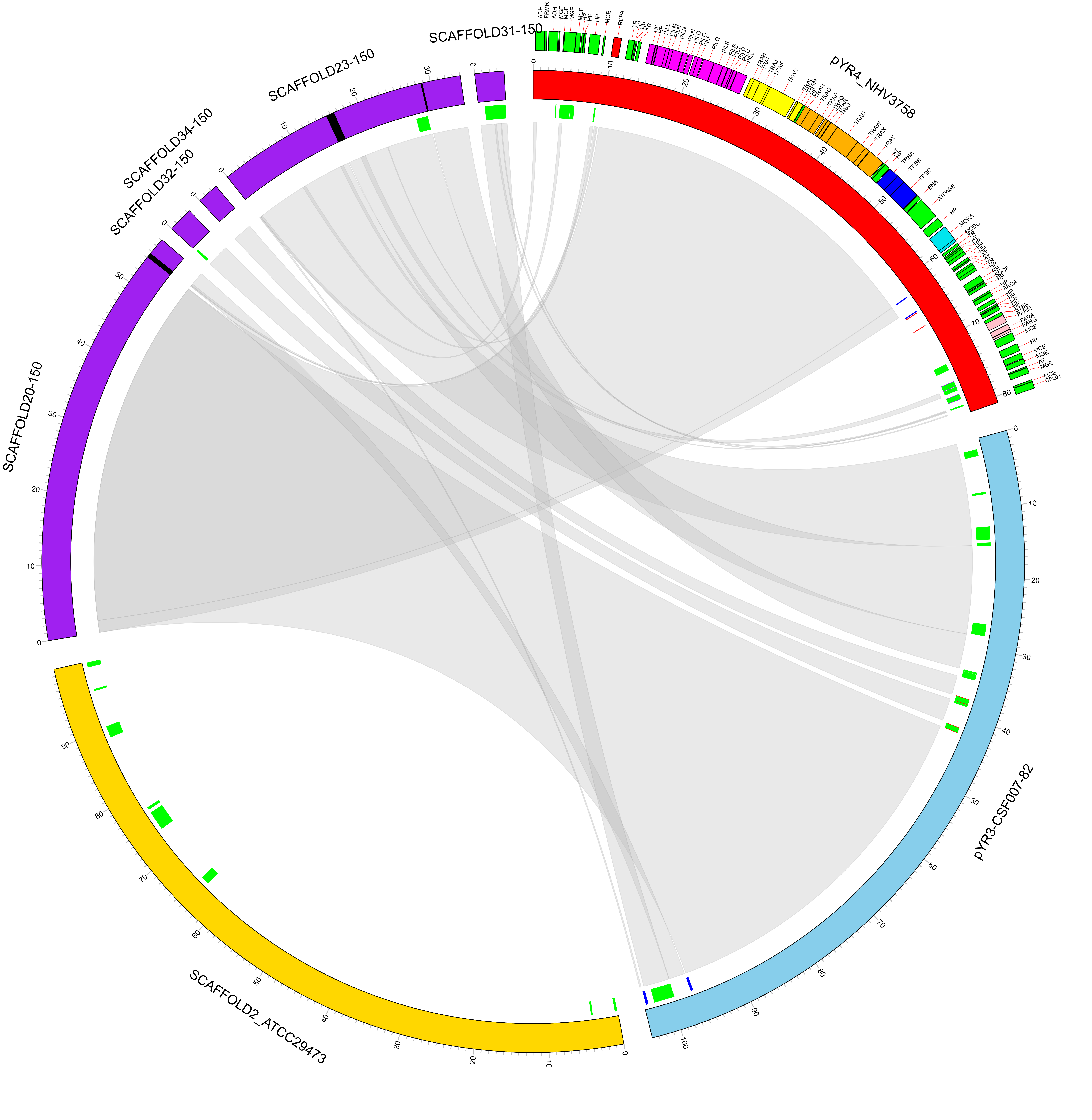

pYR4_NHV3758
pYR3-CSF007-82
SCAFFOLD2_ATCC29473
SCAFFOLD20-150
SCAFFOLD32-150
SCAFFOLD34-150
SCAFFOLD23-150
SCAFFOLD31-150

Supplementary figure 7.

Circular representation of comparative analysis as in Figure 2 showing ribbon links of 150 with the two plasmids, pYR3 and pYR4.

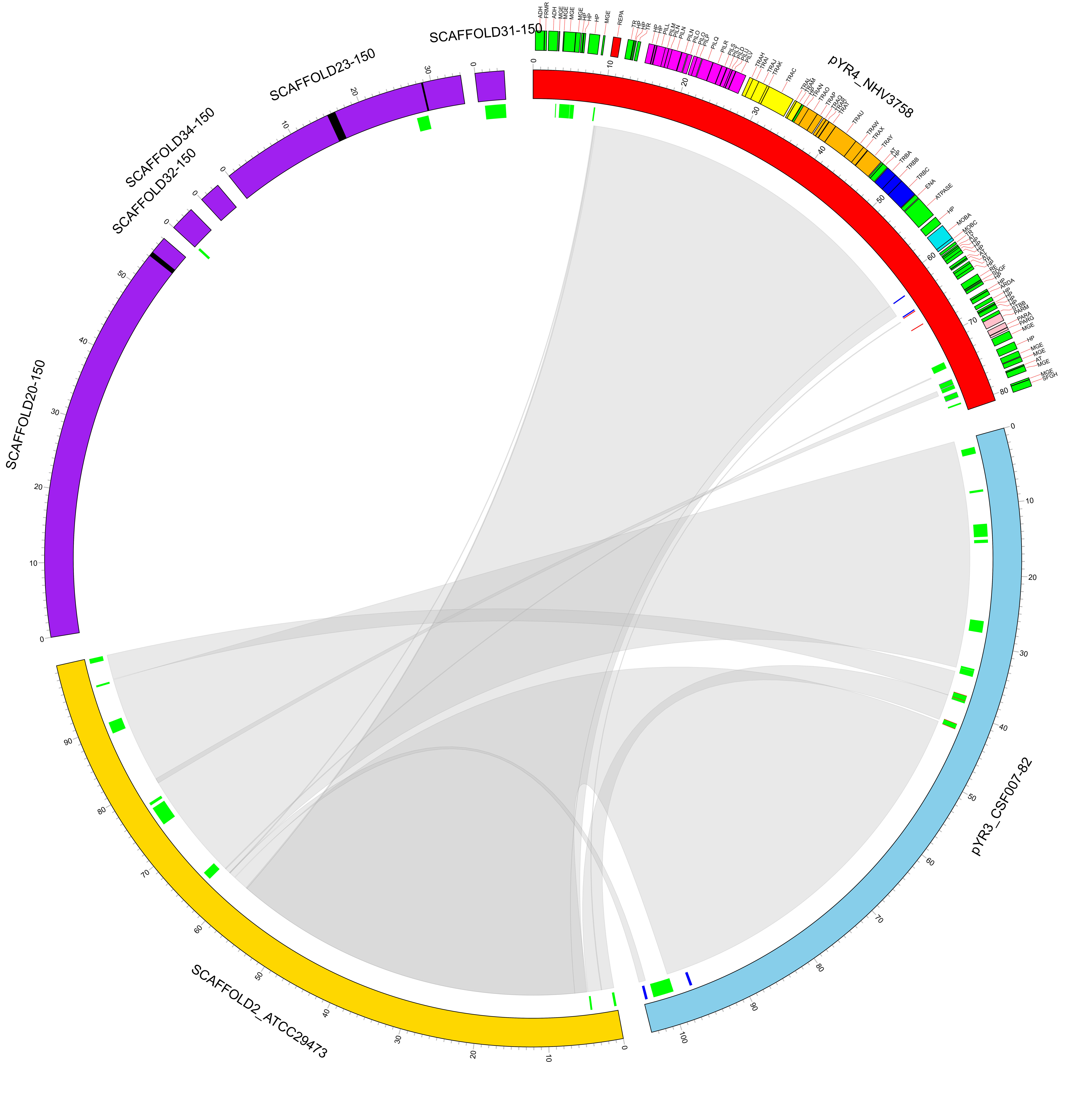

SCAFFOLD31-150
SCAFFOLD23-150
SCAFFOLD34-150
SCAFFOLD32-150
SCAFFOLD20-150
pYR4_NHV3758
pYR3_CSF007-82
SCAFFOLD2_ATCC29473
